# Lung stereotactic body radiotherapy with an MR-linac – Quantifying the impact of the magnetic field and real-time tumor tracking

**DOI:** 10.1016/j.radonc.2016.04.019

**Published:** 2016-06

**Authors:** Martin J. Menten, Martin F. Fast, Simeon Nill, Cornelis P. Kamerling, Fiona McDonald, Uwe Oelfke

**Affiliations:** Joint Department of Physics at The Institute of Cancer Research and The Royal Marsden NHS Foundation Trust, London, UK

**Keywords:** MR-linac, MR-guided radiotherapy, Real-time adaptive radiotherapy, Tumor tracking, Lung stereotactic body radiotherapy

## Abstract

**Background and purpose:**

There are concerns that radiotherapy doses delivered in a magnetic field might be distorted due to the Lorentz force deflecting secondary electrons. This study investigates this effect on lung stereotactic body radiotherapy (SBRT) treatments, conducted either with or without multileaf collimator (MLC) tumor tracking.

**Material and methods:**

Lung SBRT treatments with an MR-linac were simulated for nine patients. Two different treatment techniques were compared: conventional, non-tracked deliveries and deliveries with real-time MLC tumor tracking, each conducted either with or without a 1.5 T magnetic field.

**Results:**

Slight dose distortions at air-tissue-interfaces were observed in the presence of the magnetic field. Most prominently, the dose to 2% of the skin increased by 1.4 Gy on average. Regardless of the presence of the magnetic field, MLC tracking was able to spare healthy tissue, for example by decreasing the mean lung dose by 0.3 Gy on average, while maintaining the target dose.

**Conclusions:**

Accounting for the magnetic field during treatment plan optimization allowed for design and delivery of clinically acceptable lung SBRT treatments with an MR-linac. Furthermore, the ability of MLC tumor tracking to decrease dose exposure of healthy tissue, was not inhibited by the magnetic field.

The integration of magnetic resonance (MR) imaging with either a linear accelerator (MR-linac) or Cobalt-60 radiation sources is one of the recent advancements in radiotherapy technology [1], [2], [3]. It allows radiotherapeutic treatment of patients while simultaneously acquiring MR images featuring excellent soft-tissue contrast. However, there are concerns regarding the distortion of dose distributions caused by the MR scanner’s magnetic field deflecting secondary electrons due to the Lorentz force [4]. As this predominantly alters dose distributions at air-tissue-interfaces, treatments of lung tumors are expected to be particularly affected.

Lung tumors can exhibit deformations, rotations and translations of up to a few centimeters, which may result in underdosage of the target and additional irradiation of nearby healthy organs [5]. Techniques have been developed to recover the target dose by adapting the treatment to the tumor motion in real-time. This can be achieved by repositioning the patient using a robotic treatment couch [6], [7], tilting or moving the entire linear accelerator [8], [9] or by dynamically adapting the linear accelerator’s multileaf collimator (MLC) [10], [11], [12]. In the future, MLC tumor tracking may be performed on MR-guided radiotherapy units allowing adaptation of the treatment based on anatomical information obtained from high-contrast MR images [13], [14].

Recent studies have investigated radiotherapy of lung tumors under the influence of an external magnetic field [15], [16]. They reported dose distortions due to the magnetic field that resulted in an underdosage of the target volume. However, these studies only included one patient each and did not consider the effect of the magnetic field during optimization of the treatment plan, which has been shown to partly alleviate its distorting effect in phantom experiments and for other tumor sites [17], [18]. Furthermore, the influence of intrafractional changes of the patient anatomy was not evaluated in either study.

This planning study investigates the clinical feasibility of lung stereotactic body radiotherapy (SBRT) treatments with an MR-linac. It accounts for the magnetic field during optimization of the treatment plan and also considers intrafractional tumor motion with and without compensation by real-time MLC tumor tracking for a cohort of nine patients.

## Material and methods

### Patient cohort

Nine 4DCT scans of lung cancer patients (four male/five female, age: 69–86 years) undergoing SBRT at our institution were selected from a group of 15. The other six 4DCT scans were excluded from this study as they featured severe image artifacts in the tumor region, which might have substantially influenced the accuracy of the deformable image registration (DIR) used for dose accumulation. The mean tumor volume was 10.1 cc (range: 3.3–25.5 cc). The tumor was located in the upper left lobe of the patient’s lung in five cases and in the lower left lobe and upper right lobe in two patients each. The centroids of the tumors exhibited a mean three-dimensional peak-to-peak motion of 5.7 mm (range: 1.5–14.1 mm).

### Contouring

The tumor and organs-at-risk were delineated on the peak-exhale phase using RayStation, research version 4.6.100.12 (RaySearch Laboratories AB, Stockholm, Sweden). The peak-exhale phase was selected for contouring and treatment planning as it is usually the most reproducible phase of the breathing cycle and its images consequently feature only small motion artifacts. Normal tissue structures were outlined according to the RTOG 1021 guideline [19]. Two different approaches were used to define the target: an internal target volume (ITV) approach for *conventional* treatments without real-time motion compensation and a so-called moving target volume (MTV) approach for *tracked* deliveries with MLC tumor tracking, as suggested by Kamerling and Fast et al. [20]. The ITV is defined by the union of the gross tumor volumes (GTV) contoured on each of the 10 4DCT phases. The MTV is obtained by shifting the GTV contour of each 4DCT phase so that its centroid overlaps with the centroid of the GTV on the reference phase. Calculating the union of the shifted contours and using it as planning target implicitly accounts for tumor deformation during MLC tumor tracking, which localizes and adapts the treatment to translations of the target’s center. Use of the MTV is a conservative approach as the maximum extent of tumor deformation measured over all phases is assumed to occur in every single phase. Both the ITV and MTV were expanded by an isotropic margin of 5 mm to create the respective planning target volumes (PTV) as it is clinical practice at our institution to account for residual localization and setup errors.

### Treatment planning

The outlined contours were then transferred to the Monaco treatment planning software, research version 5.19.00 (Elekta AB, Stockholm, Sweden). The software features a machine model of the MR-linac currently being developed by Elekta. This model includes the treatment beam with a nominal beam energy of 7 MV, the MLC as well as the cryostat. Each of the 160 leaves of the MLC, which is fixed to 90° (coordinate system according to IEC-61217), has an isocenter-projected width of 7.15 mm and the maximum field size is 27.20 cm parallel to leaf direction and 57.15 cm perpendicular to leaf direction [21]. This large field of view allows treatment of peripheral tumors even though the treatment beam isocenter is fixed to the center of the bore. The cryostat is simulated by beam-angle-dependent filtration layers attenuating and scattering the treatment beam. Monaco is able to calculate doses and optimize treatment plans for a patient geometry located in a magnetic field. For each patient four different step-and-shoot IMRT treatment plans were prepared on the peak-exhale phase: two each for the ITV + 5 mm or MTV + 5 mm target volume; each either with or without a 1.5 T magnetic field oriented in the superior–inferior patient direction. In order to keep all plans as comparable as possible nine equidistant beams were used in all cases and each plan was normalized to deliver 54 Gy in 3 fractions to 95% of the respective PTV. Additionally, we aimed to deliver a similar maximum target dose. The treatment plans fulfilled the organ-at-risk constraints of the RTOG 1021 guideline in almost all cases. In two patients, in which the tumor was attached directly to the pleural wall, the constraints to the skin and ribs were violated and in one patient the ${\text{R}}_{50\%}$ dose spillage constraint to the entire patient was slightly exceeded. These guideline violations occurred independently of the used planning strategy or presence of magnetic field. All treatment plans were reviewed by an experienced radiation oncologist and would have been accepted for clinical delivery at our institution based on the planned dose distributions.

### Simulated dose delivery and dose accumulation

Delivery of the treatment plans was simulated in Monaco by calculating the dose distribution of each plan for all 10 4DCT phases. Plans were calculated using Monaco’s build-in Monte Carlo does engine based on work by Hissoiny et al. with a statistical uncertainty of 2% on a dose grid of 0.25 × 0.25 × 0.25 cm^3^ [22], [23]. For the conventional, non-tracked treatments, the isocenter and treatment field apertures were kept unchanged. For the tracked deliveries, the treatment plans were exported and post-processed in our in-house MLC tumor tracking software [12]. All segments were deformed according to the beams-eye-view target translation in each 4DCT phase and resnapped to the MLC grid to account for the finite MLC leaf width [24]. The resulting 10 treatment plans were reimported into Monaco and the dose was calculated for each phase.

Afterward, the dose distributions were transferred to RayStation. One tenth of the dose simulated to each phase was accumulated on the peak-exhale phase. The resulting dose distributions were used for the further evaluation. Dose accumulation was based on direct dose mapping and RayStation’s hybrid DIR aided by the manually delineated patient and lung contours. Although deformable dose accumulation and validation methods for its accuracy are still being heavily researched [25], [26], we believe that visual inspection of the deformation vector fields with regard to their physical plausibility and the use of a single 4DCT scan allowed for confident deployment of this methodology.

### Evaluation and statistical tools

The effect of the magnetic field was evaluated by comparing the simulated dose distributions delivered without a magnetic field to the ones delivered with a 1.5 T magnetic field. This was done for conventional treatments as well as tracked deliveries. By comparing the conventional dose distributions to the tracked ones, the dosimetric effect of MLC tumor tracking was investigated.

Due to the treatment plan optimizer having to solve a different optimization problem dependent on the PTV definition and magnetic field strength, the four treatment plans per patient could differ with regard to beam weights and fluence shapes. Therefore, we refrained from comparing the plans on a voxel-by-voxel basis. Instead, the differences in a number of dose–volume metrics as well as the integral deposited energy in the patient were evaluated. The integral deposited energy was calculated by summing over all voxels and multiplying the mass density of each voxel with the corresponding dose and voxel volume. The density grid was derived from the CT numbers and interpolated to the dose grid. Primary endpoints being tested were the differences in the dose to 98% of the GTV, dose to 2% of the skin volume, mean lung dose and the integral deposited energy. These endpoints were selected *a priori* as they were expected to be strongly influenced by a magnetic field or tumor tracking. Statistical significance of the differences was evaluated using a two-sided paired t-test after verifying that the differences were normally distributed using Lilliefors test [27]. For the primary endpoints, a significance level of *p* = 0.0125 was chosen by performing a Bonferroni correction for multiple testing for a significance level of *p* = 0.05 [28]. Differences in several other dose–volume metrics were evaluated in an exploratory analysis without correcting for multiple testing with a significance level of *p* = 0.05.

## Results

### Influence of the magnetic field

**Fig. 1:**
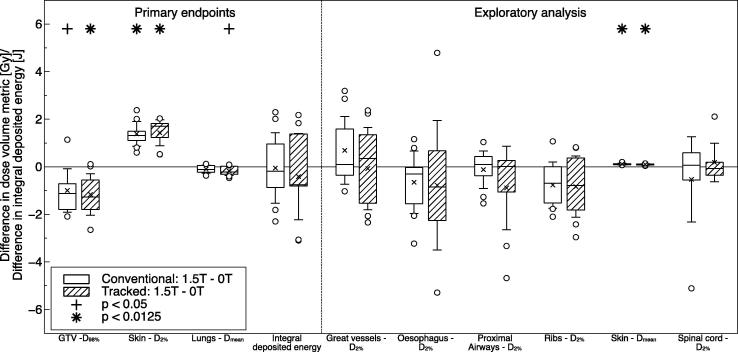
Differences in the investigated dose–volume metrics and integral deposited energy due to the presence of a 1.5 T magnetic field. The compared treatments were simulated either without tumor tracking (conventional: 1.5 T–0 T) or with MLC tumor tracking (tracked: 1.5 T–0 T). The boxes mark the first and third quartile, while the bands inside mark the median. The average values are represented by the crosses and the standard deviations by the whiskers. Outliers are denoted by the circles. All values are extrapolated from one fraction to the entire treatment.

The presence of the 1.5 T magnetic field caused significant changes in several investigated dose-volume metrics, especially those featuring air-tissue-interfaces (see Fig. 1). Most prominently, the dose to 2% of the skin volume increased significantly for treatments with as well as without MLC tracking. Dose to 98% of the GTV decreased in conventional deliveries and significantly decreased in tracked deliveries, while the mean lung dose slightly decreased. The integral deposited energy varied over all 36 plans (mean: 36.5 J, range: 19.9–62.2 J), but the magnetic field did not have a systematic effect on it. The exploratory analysis found a significant increase in the mean skin dose due to the magnetic field. The remaining dose-volume metrics did not show any significant differences. It is important to note that despite all of these effects, the GTV was covered by the prescribed dose in all 36 simulated dose deliveries. Furthermore, there were no substantial violations of normal tissue constraints, that did not already occur in the planned dose distributions (see section *Treatment planning*).

**Fig. 2:**
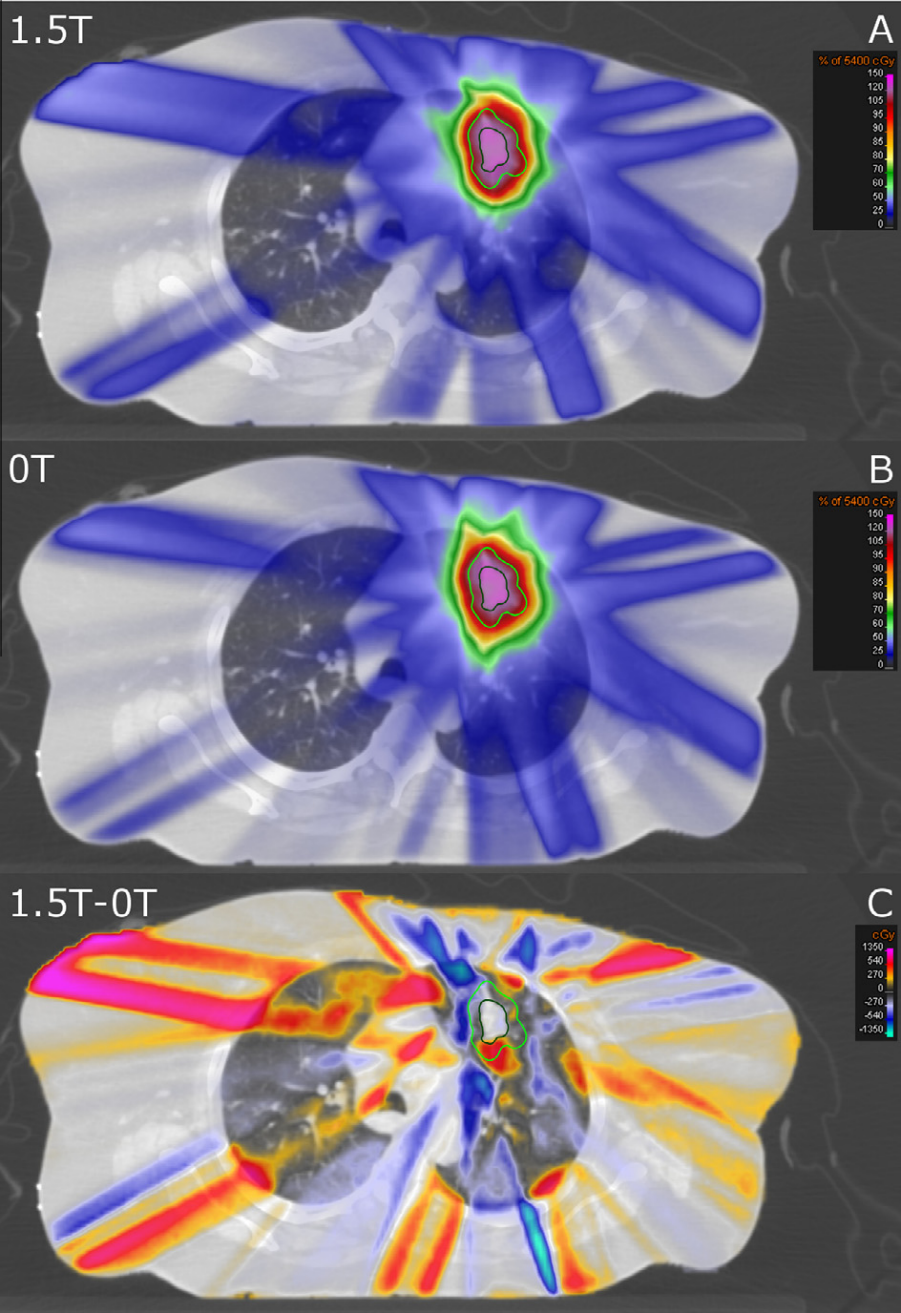
Transversal CT slice of a lung cancer patient. Overlaid are either (A) the simulated dose accumulated on the reference 4DCT phase with a 1.5 T magnetic field present, (B) the delivered dose without a magnetic field and (C) the difference (A - B) between those two distributions. The GTV is contoured in dark green and the ITV + 5 mm is contoured in light green.

An example, which highlights the observed effects, is shown in Fig. 2. At air-tissue-interfaces in the lung as well as in the skin of the patient returning electrons cause local hot spots. For this case, the dose to 2% of the skin increased from 7.4 Gy to 9.2 Gy and the mean lung dose increased from 4.2 Gy to 4.3 Gy in the presence of the magnetic field. Even though both these increases are among the highest observed in all patients, the absolute values for both metrics are about average and substantially smaller than the inter-patient variability. Averaged over all patients the dose to 2% of the skin is 8.7 Gy (range: 5.8–11.1 Gy) and the mean lung dose is 4.7 Gy (range: 3.5–6.2 Gy). The long “stripes” of locally increased or decreased dose along the beam paths are partly caused by the inverse treatment plan optimizer creating differently weighted and shaped segments depending on the optimization problem.

### Effect of MLC tumor tracking

**Fig. 3:**
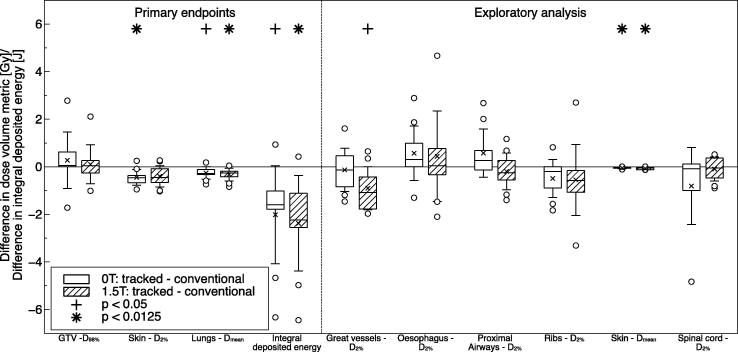
Differences in the investigated dose–volume metrics and integral deposited energy due to real-time MLC tumor tracking. The compared dose distributions were simulated either without a magnetic field (0 T: tracked–conventional) or with a magnetic field (1.5 T: tracked–conventional).

Tracked deliveries featured significant decreases in the dose to 2% of the skin volume, mean lung dose and integral deposited energy, while the dose to 98% of the GTV remained similar compared to conventional deliveries (see Fig. 3). Furthermore, the exploratory analysis found the dose to 2% of the great vessels and mean skin dose to decrease significantly. The magnitude of the differences is similar for treatments delivered at 1.5 T and 0 T. The differences are all most likely caused by the larger PTV for conventional treatments compared to tracked deliveries in cases of target motion.

**Fig. 4:**
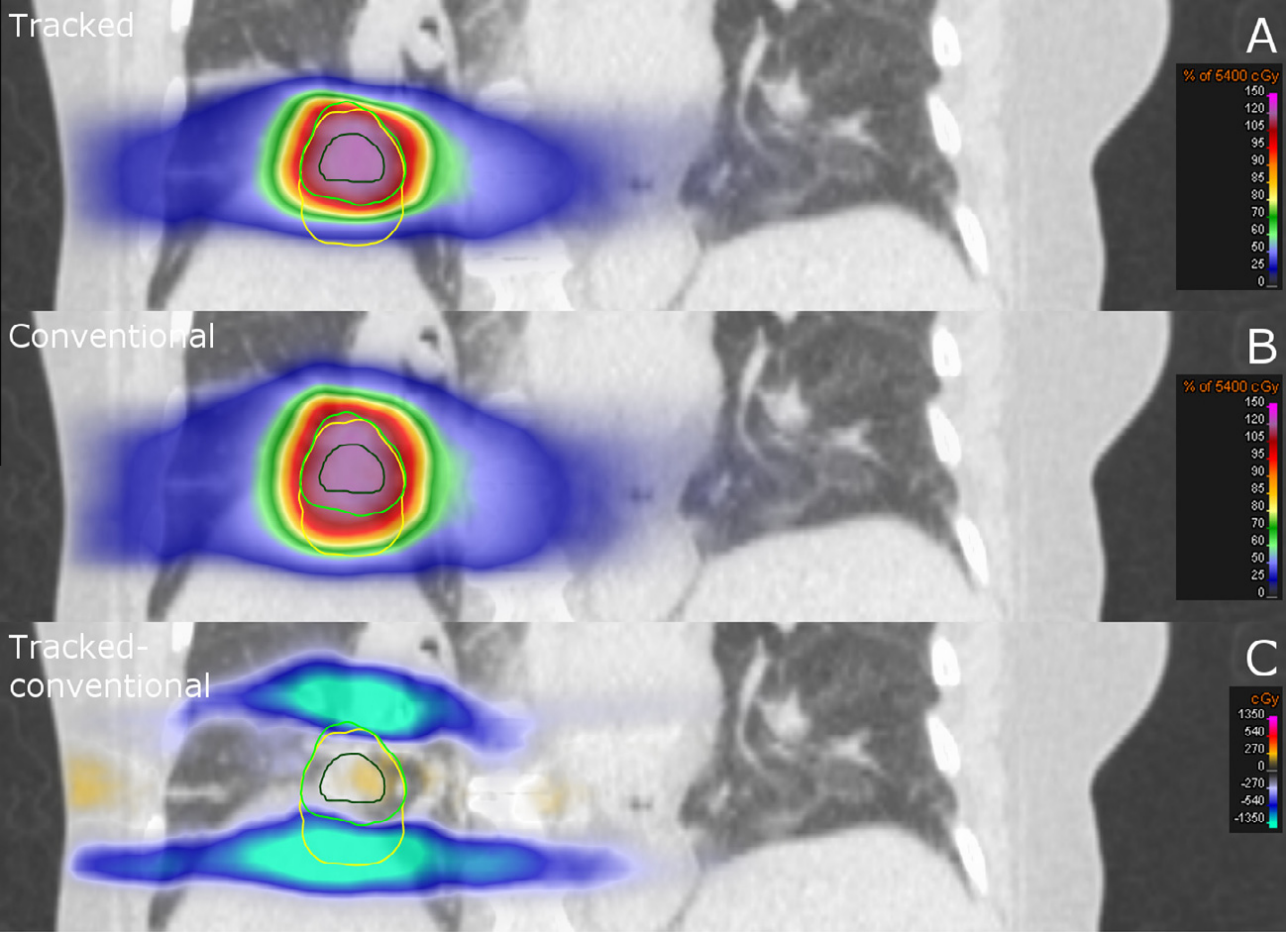
Coronal CT slice of a lung cancer patient. Overlaid are either (A) the simulated delivered dose accumulated on the reference 4DCT phase deploying MLC tumor tracking and a MTV target definition approach at 1.5 T, (B) the delivered dose using the conventional, non-tracked ITV approach at 1.5 T and (C) the difference (A - B) between those two distributions. The GTV is contoured in dark green, while the MTV + 5 mm is contoured in light green and the ITV + 5 mm in yellow.

**Fig. 5:**
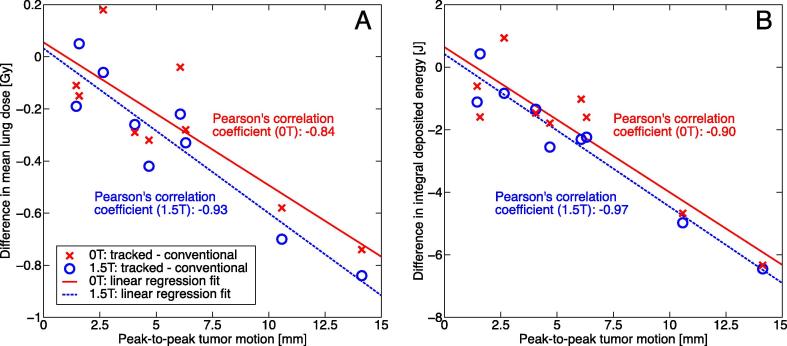
Graph presenting the differences in the (A) mean lung dose and (B) integral deposited energy due to tumor tracking dependent on the peak-to-peak tumor motion. Each data point denotes one patient case and the lines represent linear regression fits to the data.

Fig. 4 presents the dose distributions delivered at 1.5 T for the case with the largest observed peak-to-peak tumor motion of 14.1 mm. This results in the ITV + 5 mm being 44% larger than the MTV + 5 mm (22.9 versus 15.8 cc). In this case, tracking was able to reduce the mean lung dose by 0.8 Gy and the integral deposited energy by 6.4 J while maintaining the tumor dose. Both differences were the largest among all investigated cases. The strong correlation between the decrease in the mean lung dose and integral deposited energy and the observed peak-to-peak tumor motion is shown in Fig. 5.

## Discussion

This study has found that considering the effect of the magnetic field during the treatment planning stage allows to conduct clinically acceptable lung SBRT treatments with an MR-linac. The 1.5 T magnetic field did not inhibit the ability to generate treatment plans fulfilling the planning goals set by the RTOG 1021 guideline. Still, the presence of a magnetic field caused systematic differences in the dose exposure of the tumor and organs-at-risk. Most prominently, the skin dose increased with the magnetic field present. This and the observed reduction in lung dose are caused by electrons returning to the air-tissue-interface they were ejected from due to the Lorentz force. Additionally, a decrease in the dose level covering the GTV was found, although it received more than the intended dose in all investigated cases. Differences in the dose to other organs-at-risk were strongly dependant on the individual patient geometry and no general trends were found. All of these differences are small compared to the magnitude of inter-patient heterogeneity and are expected to only have a very minor clinical impact.

The ability of adequate planning to partially compensate for the effect of a magnetic field was also reported by Raaijmakers et al. [17]. They designed radiotherapy treatment plans in a magnetic field for a prostate, larynx and oropharynx cancer patient and found a slight increase in skin dose to be the most prominent effect. Van Heijst et al. also found an increase in skin dose while investigating the effect of a magnetic field on radiotherapy of breast tumors in 10 patients [29]. However, they were able to reduce this increase by using an accelerated partial breast irradiation approach instead of whole breast irradiation. Two studies investigated the effect of magnetic fields on radiotherapy of lung tumors [15], [16]. Kirkby et al. reported severe dose distortions in lung dose and a reduction in PTV coverage while evaluating the effect of a magnetic field in one lung patient. Yang et al. also warned of dose distortions at air-tissue-interfaces after investigating the effect of a magnetic field on the dose delivered with rotational therapy for single prostate, head-and-neck and lung patients. Our study has found similar dose distortions, although of substantially smaller magnitude. This is probably because neither Kirkby et al. nor Yang et al. considered the magnetic field at the stage of treatment plan optimization. Kirkby et al. focussed on the effect of different magnetic field strengths and orientations. Our study only investigated the effect of a 1.5 T magnetic field oriented along the superior–inferior patient axis as has been realized in the MR-linac prototype at University Medical Centre Utrecht [1]. This case was found to be one of the most challenging ones in the study by Kirkby et al. In our study, the observed local dose hot spots at air-tissue-interfaces did not conflict with any clinical planning goals. Still, treatment plan evaluation metrics more sensitive to the occurrence of these hot spots might be needed to evaluate the quality of treatment plans designed for delivery in the presence of a magnetic field.

Additionally, our study compared treatments with real-time MLC tumor tracking to conventional deliveries. Tumor tracking was shown to be able to maintain dose coverage of the GTV while reducing the integral deposited energy in the patient. Less deposited energy usually led to a decrease in dose to organs-at-risk, mainly the skin and normal lung tissue. The ability of real-time tumor tracking to decrease dose exposure of healthy tissue was not inhibited by the magnetic field. This was the main interest of this study when comparing tracked deliveries to conventional ones, rather than an extensive investigation of the benefits of MLC tumor tracking. Localization errors and their effect on MLC tracking were not considered in this study. Furthermore, the deployed methodology used a single 4DCT scan to both design treatment plans and simulate the delivery. This disregards anatomical changes over the course of the treatment or even a single fraction, such as baseline shifts or setup errors. It is palpable that tracked treatments would be able to more reliably achieve target dose coverage in the presence of these changes compared to conventional deliveries. The advantages of tracking might be increased even further by implementing a more advanced form of MLC tumor tracking. For example, MLC tumor tracking may be able to compensate for tumor deformation and rotation [30]. Moreover, a perceived advantage of real-time tumor tracking is the increased confidence that the dose is accurately delivered. This might encourage a reduction of treatment margins, which could lead to an additional reduction of the dose to healthy tissue [31]. In this study the PTV expansion margin was set to 5 mm for both conventional and tracked deliveries.

In summary, this study has evaluated the feasibility of treating lung cancer patients with SBRT with an MR-linac. When accounting for the 1.5 T magnetic field during treatment planning, we were able to design and simulate the delivery of clinically acceptable treatments. Furthermore, it was found that the ability of real-time tumor tracking to decrease dose exposure of healthy tissue was not inhibited by the magnetic field.

## Conflict of interest statement

The Institute of Cancer Research is part of the Elekta Atlantic MR-linac Research Consortium and we acknowledge financial and technical support from Elekta AB under a research agreement. However, the sponsors had no part in the design or execution of the study.
